# Mesoscale Mechanisms Governing the Shear Strength of Lunar Regolith: Effects of Low Confining Stress and Irregular Particle Morphology

**DOI:** 10.3390/ma19071439

**Published:** 2026-04-03

**Authors:** Jun Chen, Ruilin Li, Yukun Ji, Pinqiang Mo

**Affiliations:** 1 Institute of Mine Safety, China Academy of Safety Science and Technology, Beijing 100012, China; 2 State Key Laboratory of Intelligent Construction and Healthy Operation and Maintenance of Deep Underground Engineering, China University of Mining and Technology, Xuzhou 221116, China

**Keywords:** shear strength, lunar regolith, CUMT-1 lunar regolith simulant, irregular particle morphology, low geostatic stress, macro–mesoscopic strength correlation model

## Abstract

Understanding the mechanical behavior of lunar regolith is critical for the success of future lunar excavation and construction missions. Irregular particle morphology and low geostatic stress are recognized as key factors contributing to the high internal friction angle of this unique extraterrestrial geomaterial. However, the underlying mechanisms by which low geostatic stress enhances shear strength remain unclear, and the multiscale effects of particle morphology on shear strength evolution are not yet fully elucidated. In this study, consolidated drained triaxial compression tests were performed on CUMT-1 lunar regolith simulant and Fujian standard sand to investigate their macroscopic mechanical behavior. Complementary discrete element simulations of biaxial compression were conducted to analyze mesoscopic mechanical responses of granular materials under the influence of multiscale particle morphology and confining stress. A robust macroscopic–mesoscopic strength correlation model was established, incorporating normalized mean interparticle contact force and mean coordination number to predict the normalized deviatoric stress of granular assemblies. Based on this model, the mesoscopic mechanisms through which irregular particle morphology and low geostatic stress enhance the internal friction angle were quantitatively investigated. The findings offer new insights into the shear strength characteristics of in situ lunar regolith and provide theoretical support for lunar surface construction and excavation operations.

## 1. Introduction

The lunar regolith layer is a critical repository of mineral resources [1,2,3] and forms the primary load-bearing foundation for future lunar bases [4,5,6]. Its economic importance is far greater than that of the underlying bedrock, at least in the foreseeable future [7]. Hence, investigating the mechanical strength of the lunar regolith is essential for the success of upcoming lunar surface activities [8,9].

Previous geotechnical experience indicates that the shear strength of the lunar regolith is primarily determined by its relative density [10,11], particle size distribution [12,13], particle morphology [14,15], in situ stress [16,17], and single-particle strength [18,19]. Among these factors, the irregular particle morphology and the low geostatic stress, which arise from the special geological processes and the Moon’s gravitational field, respectively, are defining characteristics that distinguish lunar regolith from terrestrial soils [20,21,22,23,24,25,26,27]. Hence, systematically investigating the effects of both factors is crucial for understanding the mechanical behavior of lunar regolith.

The influence of particle morphology on the shear strength of lunar regolith has mainly been investigated using the DEM method. To characterize the irregular morphology, some studies [28,29] have used high particle-particle friction coefficients exceeding 1. However, this approach contradicts physical measurements, e.g., the friction coefficient of Chang’e-5 lunar samples ranges from 0.48 to 0.76 [30], and the DNA-1 lunar simulant ranges from 0.23 to 0.51 [31]. Jiang et al. [32,33,34] proposed a rolling resistance model that captures the effects of irregular particle morphology while reducing computational costs. Despite its high computational efficiency, this abstract model fails to address morphology-induced properties like soil deformation and anisotropy. Nie et al. [35,36] attempted to classify the particle morphology of lunar regolith into two categories: overall regularity and friction coefficient. Then they investigated the potential effects of these two geometrical features on the shear strength of Chang’e-5 lunar regolith [30], marking the first multi-scale study on the realistic particle shape of lunar regolith. However, the morphological classification approach adopted by Nie et al., which is based on two scales, is relatively simplified compared with more comprehensive mainstream methods. which typically include three scales [14,37,38]. As a result, some detailed findings might have been overlooked.

The influence of in situ stress on the shear strength of lunar regolith has predominantly been studied through laboratory triaxial compression tests. Within a confining pressure range of 1–2200 kPa, many lunar regolith simulants, including JSC-1 [39], JSC-1A [40], MLS-1 [41], NAO-1 [42], GRC-3 [43], and QH-E [44], have exhibited a general trend of decreasing internal friction angle with increasing confining pressure. Similarly, some terrestrial sands have demonstrated comparable mechanical behavior [45,46,47]. Hence, it is inferred that the low geostatic stress on the moon will contribute to an increase in the internal friction angle [48]. However, the mesoscopic mechanism by which in situ stress affects the internal friction angle remains unclear.

These studies indicate that, despite the established influence of irregular particle morphology and low geostatic stress on the macroscopic strength of lunar regolith, the underlying mechanisms remain insufficiently understood. This knowledge gap largely stems from the absence of a reliable and physically interpretable macro–mesoscopic strength mapping framework. The discrete element method offers great potential for quantifying the mesoscopic strength parameters of lunar regolith [49,50,51], yet a unified framework capable of revealing the effects of particle morphology and low confining stress has not been established. Early studies mainly focused on establishing simple correlations between one or more mesoscopic parameters—such as force chains, coordination number, and interparticle contacts—and macroscopic strength properties [52,53]. However, these approaches are insufficient to incorporate the coupled effects of multiple parameters into a unified macro–mesoscopic strength mapping framework. In recent years, fabric-based descriptors have been widely used to characterize the anisotropic structure of granular materials [54,55]. Although this approach effectively incorporates mesoscopic information, its abstract nature limits the interpretability of the resulting formulations. Therefore, it is essential to identify robust and physically meaningful descriptors to establish quantitative macro–mesoscopic relationships.

To systematically investigate the influencing mechanisms of particle morphology and in situ stress on the shear strength of lunar regolith, consolidated drained triaxial compression tests on CUMT-1 lunar regolith simulant and Fujian standard sand were first conducted, and the complementary biaxial compression tests were performed using the DEM method for mesoscopic analysis. Based on theoretical assumptions, model formulation, and validation, a robust dimensionless model was proposed to link macroscopic and mesoscopic strength, which was then used to describe the mechanisms by which irregular particle morphology and low geostatic stress increase the internal friction angle of lunar regolith.

## 2. Material and Methods

### 2.1. Laboratory Triaxial Compression Test

#### 2.1.1. CUMT-1 Lunar Regolith Simulant and Fujian Standard Sand

**Figure 1:**
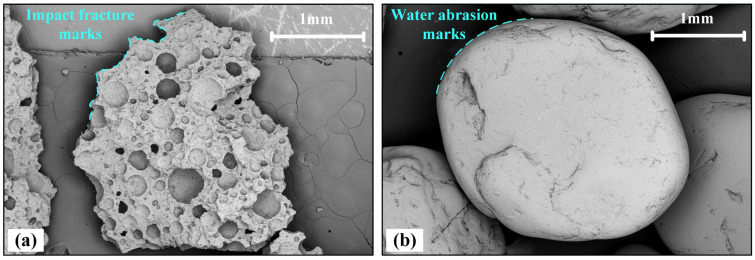
SEM images of CUMT-1 lunar regolith simulant and Fujian standard sand (FS): (**a**) CUMT-1 lunar regolith (Mag = 30×); (**b**) Fujian standard sand (Mag = 30×).

The CUMT-1 lunar regolith simulant and Fujian standard sand were used to represent lunar and terrestrial soils, respectively, as shown in Figure 1. The CUMT-1 lunar regolith simulant was developed for geotechnical use [27]. Its main components are volcanic ash and ferroferric oxide (Fe_3_O_4_). The production process involves two core techniques: high-temperature sintering and impact crushing, which could simulate the meteorite impact during the formation of in situ lunar regolith. The CUMT-1 lunar regolith simulant exhibits abundant sharp edges and textures, resembling the lunar regolith samples returned by the Chang’e-5 mission [26]. In contrast, Fujian standard sand exhibits a more rounded morphology with distinct characteristics of water erosion. Fujian standard sand was selected because it is widely used in laboratory testing and can effectively represent the typical geometric characteristics of terrestrial soils. Through comparative analysis, this enables the identification of mechanical behaviors induced by the irregular particle morphology of lunar regolith.

**Figure 2:**
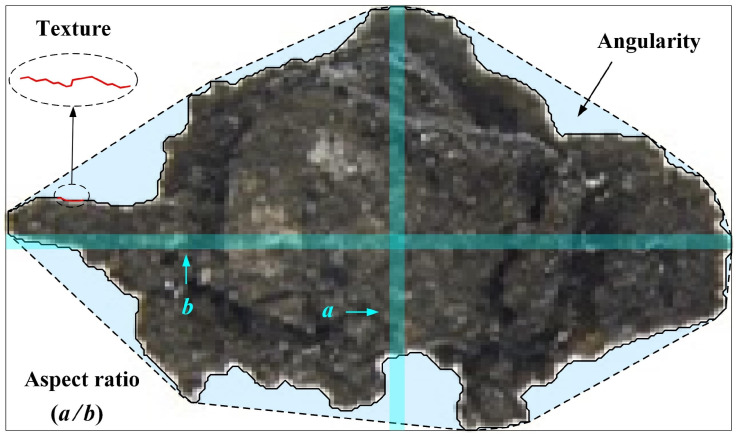
Particle morphology at three observation scales (illustrated by a typical lunar regolith particle from the Chang’e-5 mission).

Particle morphology exhibits multi-scale properties. It is typically divided into three scales: overall, local, and detailed geometric features [37]. However, the specific quantitative indicators used for each scale may vary among researchers [14,38]. In this study, the aspect ratio (*Ar*), angularity (*Ag*), and texture (*Te*) are utilized to quantify the overall, local, and detailed geometric features, respectively, as shown in Figure 2. The specific definitions of these indicators can be found in previous studies [56].

The quantification results of the two types of soils are shown in Table 1. It can be observed that the particle morphology of both the CUMT-1 lunar regolith simulant and Fujian standard sand varies significantly with particle size, but the variation in the Fujian standard sand is more pronounced. Besides, the mean texture, angularity, and aspect ratio of the CUMT-1 lunar simulant are 2.14, 1.97, and 0.95 times those of the Fujian standard sand, respectively. This indicates that the particle morphology of the CUMT-1 lunar regolith simulant is more complex across all three scales than Fujian standard sand.

#### 2.1.2. Experimental Scheme

The GDS triaxial apparatus was used for laboratory triaxial tests. Its confining pressure controller has a range of 2 MPa and a resolution of 0.1 Pa, ensuring accurate pressure control under low confining stress conditions. The specimen size was Φ 39.1 × 80 mm, with a latex membrane thickness of 0.3 mm. The loading rate was set to 0.8 mm/min, and loading was terminated when the axial strain reached 15%. The specimens were dried at 105 °C before testing to simulate the absolute dry state of in situ lunar regolith. To ensure the uniformity of the specimen, the UCM method [57] was used for compaction. The specimens were consolidated under isotropic pressure for 1 min to stabilize the confining pressure before loading. Three parallel tests were performed for each experimental condition, and the average value was used to represent the final result.

**Figure 3:**
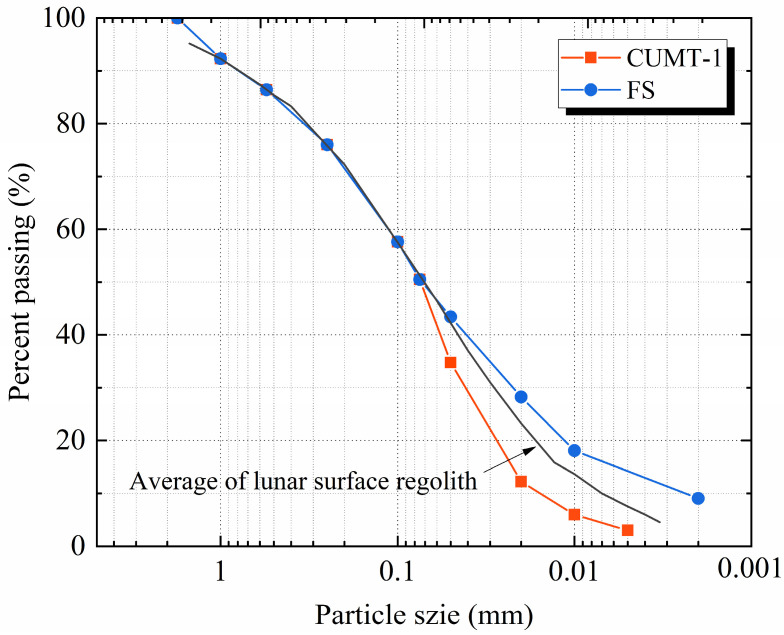
Particle size distributions of the CUMT-1 lunar regolith simulant and Fujian standard sand.

Six confining pressure conditions were designed: 10 kPa, 20 kPa, 30 kPa, 50 kPa, 100 kPa, and 150 kPa. These confining pressure levels approximately correspond to the in situ stress conditions of lunar regolith at depths ranging from about 3 to 50 m under lunar gravity. Lower confining pressures were not considered, as preliminary tests indicated that membrane effects become increasingly significant with decreasing confining pressure, which may adversely affect the accuracy of the experimental results. The relative density was 92%, representing the high compaction state of in situ lunar regolith below the lunar surface [58]. The particle size distribution (PSD) was set to the mean value of the surface lunar regolith collected by [13], as shown in Figure 3.

### 2.2. Biaxial Compression Test by DEM

#### 2.2.1. Model Parameters

The linear contact model was adopted in the DEM model, implemented using the PFC 5.0 software. The rationality of model parameters determines the reliability of simulation results. Due to computational limitations, DEM models cannot yet fully replicate the details of physical experiments, necessitating simplifications in sample size, particle morphology, particle size distribution, load boundary, and contact models. Under these asymmetrical mapping conditions, achieving mechanical behavior consistent with physical experiments typically requires iterative parameter adjustments. Based on the basic parameters proposed by Jiang et al. [59], further calibration was performed, and the final model parameters are summarized in Table 2. Based on the findings of Thornton et al. [60], increasing the particle density in a quasi-static DEM model does not adversely affect the fundamental mechanical behavior. Therefore, the particle density was scaled up by 10,000 times to enhance computational efficiency. In DEM, the timestep is determined by the minimum particle size and the Rayleigh wave speed. For realistic particle properties, the timestep is extremely small, making quasi-static simulations computationally prohibitive. Density scaling enlarges the timestep while leaving the forces, displacements, stresses, and strains of the system unchanged, only reducing particle velocities and accelerations. Since the present study focuses on quasi-static shear behavior, where inertial effects are negligible, the impact of density scaling on the mechanical response can be reasonably ignored. This method has also been employed in DEM simulations of quasi-static granular materials by Jiang et al. [59].

The gravitational field was set to zero during both consolidation and loading processes.

#### 2.2.2. Numerical Scheme

**Figure 4:**
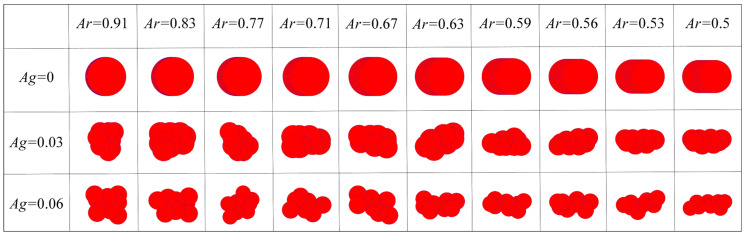
Morphologies of numerical particles in the DEM biaxial compression tests.

**Particle morphology**. Based on the quantification results in Section 2.1.1, nine groups of numerical particles with different shapes were set to investigate the influence of particle morphology. The angularity was set to three levels, 0, 0.03, and 0.06, representing non-angular particles, Fujian standard sand, and CUMT-1 lunar regolith simulant, respectively, as shown in Table 1. To ensure computational efficiency, the texture was indirectly represented by the friction coefficient, with values set to 0.24, 0.48, and 0.76. The 0.24 represents terrestrial sand [61,62], while 0.48 and 0.76 represent the lower and upper limits of the Chang’e-5 lunar regolith sample [30], respectively. Considering the negligible aspect ratio difference between CUMT-1 lunar regolith simulant and Fujian standard sand, the aspect ratio was uniformly distributed between 0.5 and 0.91. The clump was used to reconstruct the overall and local morphology of the numerical particle, with each consisting of eight balls, as shown in Figure 4. When studying the influence of particle morphology, the confining pressure in the biaxial tests was uniformly set to 50 kPa.

**Confining pressure**. Seven confining pressures were set in the biaxial tests: 5, 10, 20, 30, 50, 100, and 150 kPa. The 10–150 kPa conditions match those in the physical triaxial tests. The 5 kPa was set only in the biaxial tests due to instability observed in the physical triaxial tests at this pressure. When studying the influence of confining pressure, two numerical particle morphologies were selected: virtual CUMT-1 lunar regolith simulant (*μ* = 0.48, *Ag* = 0.06) and standard sand (*μ* = 0.24, *Ag* = 0.03).

**Figure 5:**
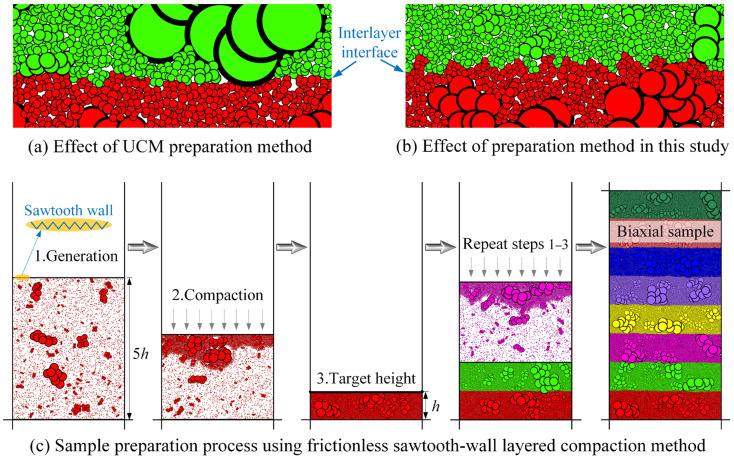
Effectiveness of the frictionless sawtooth-wall layered compaction method and its sample preparation process.

**Sample preparation**. The uniformity of samples significantly affects experimental results. Conventional methods face challenges in uniformly compacting numerical particles with irregular morphology, as shown in Figure 5a. Hence, the frictionless sawtooth-wall layered compaction method was proposed. Its key step involves using sawtooth walls to create interlocking structures for irregular particles and setting all friction coefficients to zero to promote particle interlocking. The effectiveness of this method is demonstrated in Figure 5b, where the particles in adjacent layers interlock more tightly than the UCM method. The sample preparation process using this method is illustrated in Figure 5c.

## 3. Results and Discussion

### 3.1. Triaxial Test Results

**Figure 6:**
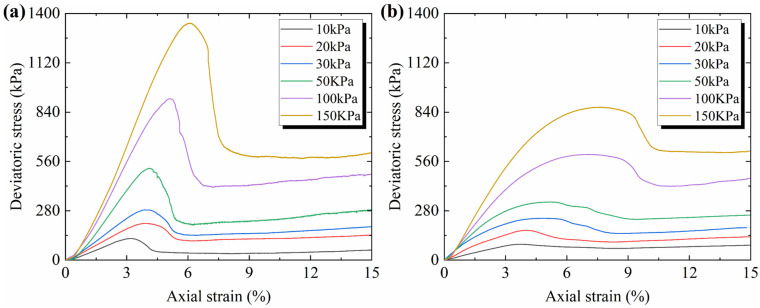
Deviator stress versus axial strain relationships of CUMT-1 lunar regolith simulant and Fujian standard sand: (**a**) stress–strain curves of CUMT-1; (**b**) stress–strain curves of FS.

The stress–strain curves of the CUMT-1 lunar regolith simulant and Fujian standard sand are presented in Figure 6. It can be observed that under the same confining pressure, the CUMT-1 lunar regolith simulant exhibits significantly higher peak stress and more pronounced strain-softening behavior. In contrast, the residual stress difference between the two materials is minimal. Specifically, under 10–100 kPa confining pressures, the residual stress of the CUMT-1 lunar regolith simulant is slightly higher, except under 150 kPa confining pressure, where the residual stress is marginally lower than that of the Fujian standard sand. This suggests that particle morphology has little effect on residual stress compared to peak stress.

Based on the stress–strain and volume–strain curves, key shear strength parameters of both materials were obtained and summarized in Table 3. The peak friction angle and residual friction angle were calculated by

(1) $$\frac{1}{2}(\sigma_{1}-\sigma_{3})=c\cos\varphi +\frac{1}{2}(\sigma_{1}+\sigma_{3})\sin\varphi$$

where σ_1_, σ_3_, *c*, and *φ* denote the axial stress, confining stress, cohesion, and internal friction angle, respectively. Given that some stress–strain curves lack a distinct minimum point in the residual stage, the residual friction angle was uniformly calculated using the mean axial stress from the residual stage.

The dilation angle was calculated by

(2) $$\left\{\begin{array}{l} \sin\theta =-\delta \varepsilon_{p}/\delta \varepsilon_{q}\\ \delta \varepsilon_{p}=-\delta V/V\\ \delta \varepsilon_{q}=-\delta l/l+\delta V/3V \end{array}\right.$$

where *ε_p_* and *ε_q_* represent the volumetric and shear strain increments, respectively, while *V* and *l* represent the volume and height of the sample, respectively. The interval from the endpoint of volumetric contraction to the peak stress point is used to calculate the maximum dilation angle. This definition could reduce subjectivity and improve the consistency of the calculation.

Based on Table 3 and Figure 6, it can be observed that as the confining pressure decreases, both the peak friction angle and residual friction angle of the two materials generally increase, and the peak stress occurs earlier. This suggests that the influence of confining pressure on soil particles with different morphologies may follow a similar trend. Such confining pressure-dependent behavior has also been clearly reported in other lunar regolith simulants, including JSC-1 [39], JSC-1A [40], MLS-1 [41], GRC-3 [43], and QH-E [44]. Furthermore, under the same confining pressure, the three strength parameters of the CUMT-1 lunar regolith simulant are generally higher than those of other geotechnical lunar regolith simulants, which can be attributed to its more complex particle morphology, as described in Section 2.1.1.

**Figure 7:**
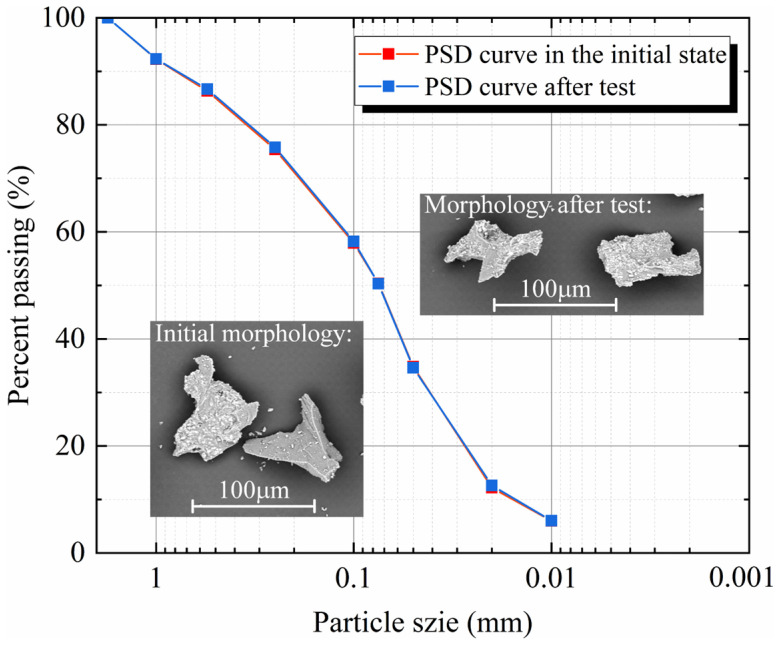
PSD curves of CUMT-1 lunar regolith simulant before and after test (σ_3_ = 150 kPa).

Considering that the particle morphology of CUMT-1 lunar regolith simulant is extremely irregular, a quantitative analysis of its grain breakage was performed, as shown in Figure 7. The particle size analysis illustrates that the particle breakage in the CUMT-1 lunar regolith simulant is negligible, as evidenced by the nearly overlapping PSD curves. The SEM images of the CUMT-1 lunar regolith simulant before and after the test also suggest that its main sharp corners and edges are well-preserved. Considering that 150 kPa corresponds to the vertical stress at a depth of approximately 47.2 m below the lunar surface (assuming a bulk density of 1960 kg/m^3^ and gravitational acceleration of 1.625 m/s^2^), it can be inferred that, under static loading conditions, irregular particle morphology is unlikely to cause significant fragmentation of shallow lunar regolith, unless the internal cementation within individual particles is weak.

### 3.2. Biaxial Test Results

**Figure 8:**
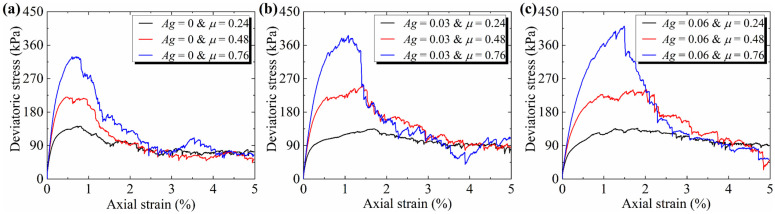
Stress–strain curves of numerical particles with different morphologies under 50 kPa confining pressure conditions: (**a**) *Ag* = 0, *μ* ∈ (0.24, 0.76); (**b**) *Ag* = 0.016, *μ* ∈ (0.24, 0.76); (**c**) *Ag* = 0.07, *μ* ∈ (0.24, 0.76).

The biaxial compression test results of the numerical particles with different particle morphologies are shown in Figure 8. It can be seen that the increases in both friction coefficient and angularity enhance shear strength and lead to more pronounced strain-softening behavior, with the friction coefficient having a greater impact. The peak stress occurs earlier with higher angularity and lower friction coefficients. Additionally, the residual stresses across all curves are similar, indicating that the residual strength of the biaxial samples exhibits low sensitivity to particle morphology. This observation is consistent with the phenomenon observed in the triaxial tests, as shown in Figure 6.

**Figure 9:**
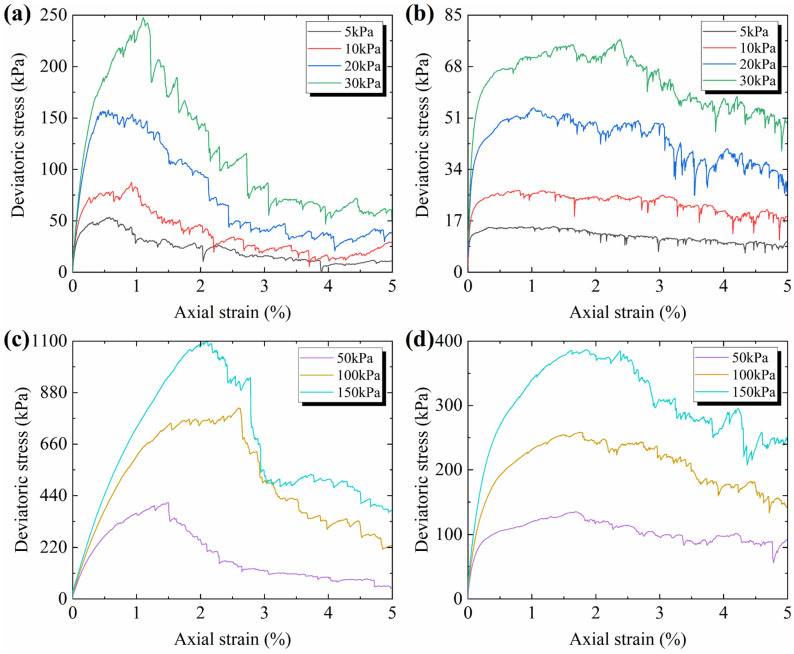
Stress–strain curves of numerical particles under different confining pressure conditions: (**a**) stress–strain curves of CUMT-1 (5–30 kPa); (**b**) stress–strain curves of FS (5–30 kPa); (**c**) stress–strain curves of CUMT-1 (50–150 kPa); (**d**) stress–strain curves of FS (50–150 kPa).

Figure 9 shows the biaxial test results of the CUMT-1 lunar regolith simulant and Fujian standard sand under 5–150 kPa confining pressures. The shear strength of the CUMT-1 lunar simulant is significantly higher than that of Fujian standard sand under the same confining pressure, along with more pronounced strain-softening behavior. Besides, the peak strength of both granular assemblies occurs earlier as the confining pressure decreases. These observations are consistent with the physical triaxial test results of the CUMT-1 lunar regolith simulant, as shown in Figure 6. They are also in agreement with the overall stress–strain behavior reported for other lunar regolith simulants, such as JSC-1 [39], JSC-1A [40], and QH-E [44]. This consistency indicates that the present simulations can effectively reproduce the mechanical characteristics of lunar regolith. It is also observed that the stress–strain curves of both granular assemblies exhibit greater fluctuations. This is primarily attributed to the limitations in particle number and size distribution, which could not fully replicate the status of the physical tests.

The strength parameters of all the granular assemblies were calculated using Equations (1) and (2), and the results are presented in Table 4. The residual friction angle was calculated using the mean value of the residual stress within the axial strain range of 3% to 5%. It can be observed that both numerical CUMT-1 and numerical standard sand exhibit an increasing trend in peak friction angle as the confining pressure decreases, consistent with the results of physical tests for the two materials presented in Table 3. However, the residual strength of both materials in the biaxial tests is less sensitive to confining pressure compared to the triaxial tests. This is likely due to the limited number of particles in the biaxial tests, which reduces the effective pressure exerted by the confining pressure on the shear plane during the residual stage.

Notably, while biaxial tests show trends similar to triaxial tests in how confining pressure and particle morphology affect shear strength, significant quantitative discrepancies persist. These differences stem from computational limitations, as the DEM methods require simplifications in spatial dimension, particle morphology, particle gradation, and contact models. Under such limited simulation conditions, it is impossible to achieve consistency between numerical simulations and physical experiments across all 14 scenarios (seven confining pressures and two morphologies) by iteratively adjusting microscopic parameters in Table 2. Considering this inherent limitation, this study only analyzes the effects of confining pressure and particle morphology, without pursuing complete correspondence between DEM simulation and experimental results.

### 3.3. Discussion

#### 3.3.1. Mesoscale Influence Mechanisms of Particle Morphology

Establishing a reliable mapping relationship between mesoscopic parameters and macroscopic strength is crucial for interpreting the influence mechanism of particle morphology on shear strength. By performing a quantitative analysis of two microscopic parameters, the mean interparticle contact force and the mean coordination number, a new indicator named the normalized mean mesoscopic strength was proposed to map the macroscopic strength. Based on this indicator, the influence mechanism of particle morphology was interpreted.

**Mean interparticle contact force**

**Figure 10:**
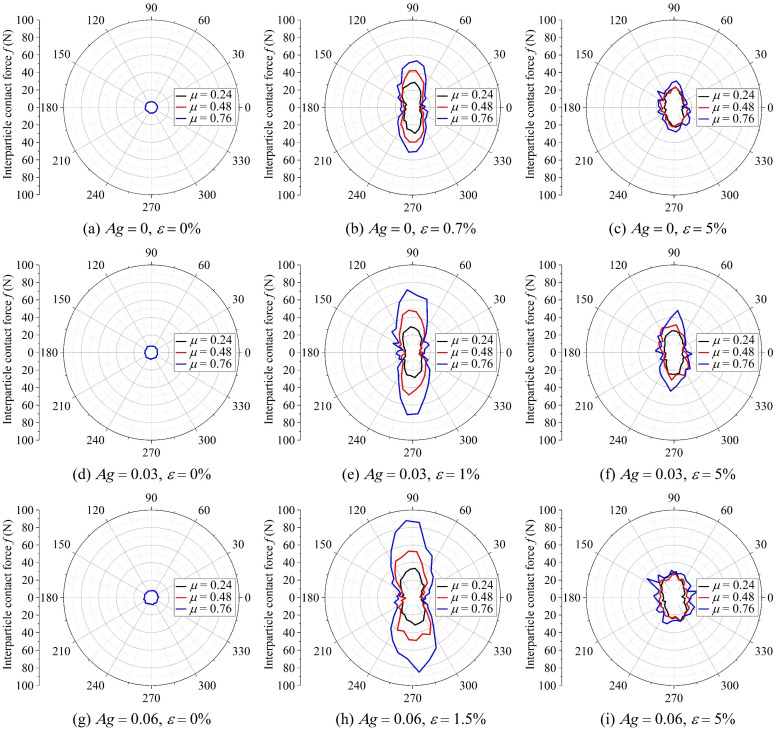
Distributions of interparticle contact forces in nine granular assemblies with different particle morphologies at various loading stages.

When a granular assembly is subjected to external loading, interparticle contact forces are generated to resist the applied load. These contact forces mainly arise from friction and interlocking among particles, making the particle morphologies that provide these forces the fundamental mesoscopic units that determine the macroscopic strength. The resultant of interparticle frictional and interlocking forces can be represented by the interparticle contact force *f* in the DEM model, and its relationship with particle morphology at different loading stages is shown in Figure 10.

In the initial stage, the magnitudes of interparticle contact forces in all the granular assemblies are very close, showing an isotropic distribution. Statistical results indicate that the interparticle contact forces are dominated by normal stress, with shear stress slightly increasing with higher friction coefficient and angularity. Under isotropic loading conditions, regardless of the complexity of the particle morphology, the interlocking and frictional structures can easily resist external stress, making it difficult to observe the contribution of particle morphology to interparticle contact forces.

As the axial strain increases, the potential of the interlocking and frictional structures among particles is gradually released, causing the interparticle contact forces to rise continuously. This is reflected macroscopically as a continuous increase in deviatoric stress. When the axial strain reaches the critical point, the interlocking and frictional structures among particles reach their maximum capacity, and the resulting interparticle contact forces reach their highest level, with the most pronounced anisotropy, taking on a peanut-shaped distribution. At this point, the granular assemblies with higher friction coefficients and angularities exhibit greater interparticle contact forces, corresponding to higher peak deviatoric stress, as shown in Figure 9. This indicates that particle morphology is a key factor determining the capacity of the interlocking and frictional structures.

When the axial strain enters the residual stage, the interparticle contact forces of granular assemblies with different morphologies converge to similar levels. This indicates that morphology is no longer the dominant factor influencing the interlocking and frictional forces among particles in this stage.

**Figure 11:**
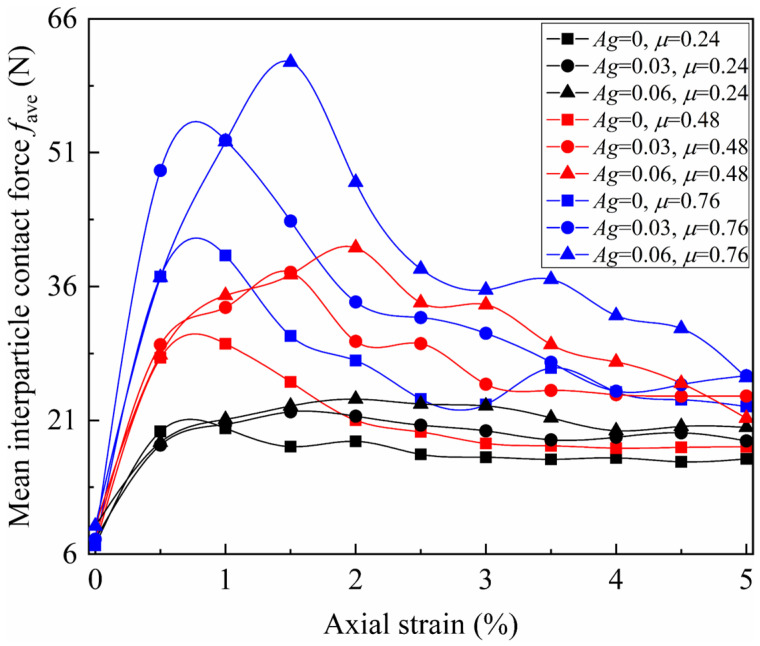
Evolution of mean interparticle contact force with axial strain.

To more intuitively quantify the influence of particle morphology, the interparticle contact forces within each granular assembly were statistically averaged by the following formula

(3) $$f_{\mathrm{ave}}=\frac{1}{N_{c}}\sum_{i=1}^{N_{c}}f_{i}$$

where *f*_ave_ represents the mean interparticle contact force, *N*_c_ represents the number of clump–clump contact points, *f*_i_ represents the interparticle contact force at the *i*-th contact points. The evolution of *f*_ave_ with axial strain is presented in Figure 11.

In the isotropic consolidation stage, the mean interparticle contact forces of the nine granular assemblies are similar, ranging from 6.9 to 9.2 N. The increase in angularity leads to a slight rise in *f*_ave_, while the friction coefficient has no effect. At the peak stage, the increases in both friction coefficient and angularity lead to a rise in the *f*_ave_, with their effects mutually reinforcing. Specifically, as angularity increases, the increment in peak strength due to the same rise in friction coefficient becomes larger. Likewise, as the friction coefficient increases, the increment in peak strength due to the same increase in angularity also becomes more pronounced. Besides, the increase in angularity and decrease in the friction coefficient both delay the peak strain. In the residual stage, the sensitivity of the *f*_ave_ to particle morphology significantly decreases, with its value in different granular assemblies approaching a similar level and overlapping partially.

It is worth noting that, although the trend of the mean interparticle contact force with axial strain closely resembles that of macroscopic deviatoric stress, it cannot accurately map the variation in macroscopic strength. For instance, the *f*_ave_ at the peak state for the granular assembly with *Ag* = 0.06 and *μ* = 0.76 is approximately 46% higher than the granular assembly with *Ag* = 0 and *μ* = 0.76, whereas the corresponding macroscopic peak deviatoric stress is only about 25% higher. To explain this phenomenon, the mean coordination number needs to be further considered.

**Mean coordination number**

The mean coordination number (*CN*) represents the density of interparticle contact forces. By iterating through the number of clump–clump contact points (*N*_c_) and the number of clumps (*N*_p_) within each granular assembly, the mean coordination number is calculated as

(4) $$CN=\frac{N_{c}}{N_{p}}$$

**Figure 12:**
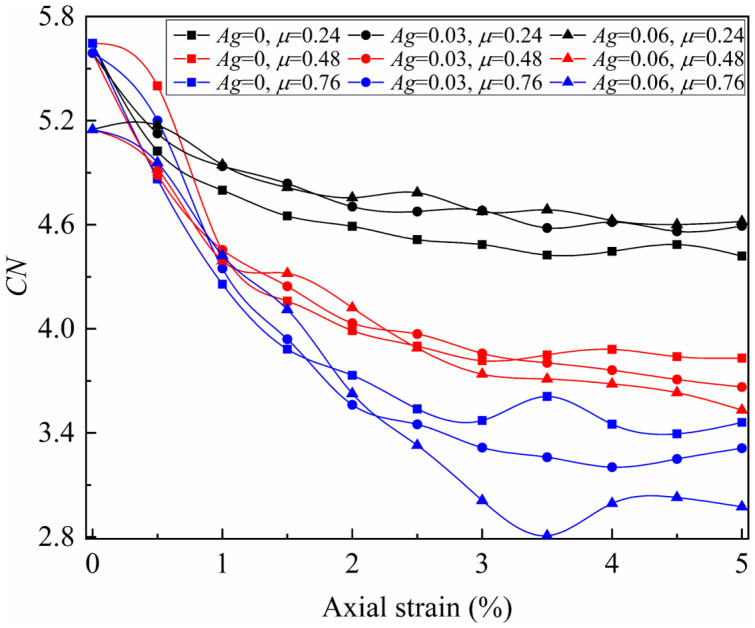
Evolution of mean coordination number with axial strain.

The mean coordination number for the nine granular assemblies at different loading stages is shown in Figure 12.

During the isotropic consolidation stage, the *CN* for the nine granular assemblies is concentrated between 5.1 and 5.7, showing no correlation with the friction coefficient and a slight decrease with increasing angularity. During the initial loading stage, all granular assemblies exhibit a rapid decline in *CN*, followed by a gradual stabilization in the residual stage. It was observed that granular assemblies with a higher friction coefficient exhibited a larger decrease in *CN*, and required more axial strain to reach stability. At the residual phase, the influence of angularity on *CN* is less pronounced than that of the friction coefficient and is significantly affected by the friction coefficient. Specifically, when the friction coefficient is 0.24, higher angularity results in a higher *CN*, while at a friction coefficient of 0.76, the opposite trend is observed. In contrast, the overall trend of friction coefficient’s effect on *CN* remains unchanged regardless of angularity.

**Normalized macroscopic and mesoscopic strengths**

For the biaxial loading condition, the external force can be decomposed into the hydrostatic stress generated by the confining pressure and the deviatoric stress generated by the axial pressure. Quantitative results indicate that adjacent particles only need to form normal forces to resist the hydrostatic stress, as shown in Figure 10a,d,g. In this case, even replacing irregular particles with smooth balls would form a stable force-chain network, meaning that the formation of hydrostatic stress is independent of the interlocking and frictional structures. In contrast, the evolution of interparticle contact force and deviatoric stress with axial strain follows a highly similar pattern, as shown in Figure 8 and Figure 11. Therefore, when establishing the macro–mesoscopic strength correlation model, deviatoric stress can be used to represent the macroscopic strength. The mesoscopic strength (*f*_mes_) can be approximately reflected by the mean interparticle contact force and the mean coordination number, as expressed by the following equation

(5) $$f_{\mathrm{mes}}=f_{\mathrm{ave}}\cdot \frac{N_{c}}{N_{p}}$$

where *f*_mes_ is the mesoscopic strength, physically defined as the mean supporting strength surrounding each particle in the granular assembly.

The macroscopic and mesoscopic strengths are normalized using the confining pressure as an intermediate quantity. The *f*_mes_ can be normalized as

(6) $${\hat{f}}_{\mathrm{mes}}={\hat{f}}_{\mathrm{ave}}\cdot \frac{N_{c}}{N_{p}}$$

where ${\hat{f}}_{\mathrm{ave}}$ represents the normalized mean interparticle contact force, calculated as

(7) $${\hat{f}}_{\mathrm{ave}}=\frac{f_{\mathrm{ave}}}{\sigma_{3}\cdot h_{0}}$$

where *h*_0_ is the initial height of the wall. Considering that the axial strain is within 5%, it is assumed that *h*_0_ remains constant.

The normalization of the macroscopic strength can be achieved as

(8) $${\hat{\sigma }}_{d}=\frac{\sigma_{1}-\sigma_{3}}{\sigma_{3}}$$

**Figure 13:**
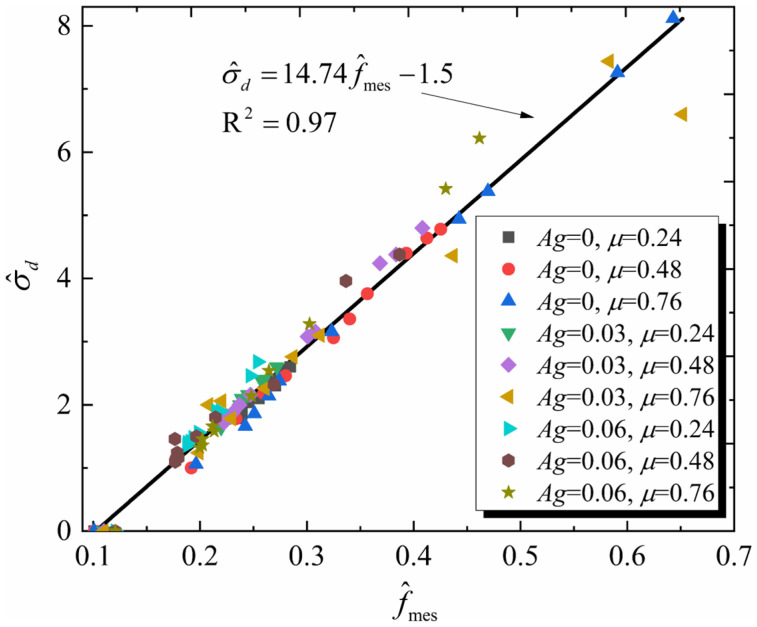
Distribution of normalized macroscopic and mesoscopic strengths in nine granular assemblies.

The values of ${\hat{f}}_{\mathrm{mes}}$ and ${\hat{\sigma }}_{d}$ in nine granular assemblies were recorded with an axial strain interval of 0.5%. A total of 99 data points were collected, with 11 evenly selected from each particle morphology conditions, covering the entire loading stage process. The distribution of these two indicators is shown in Figure 13.

It can be observed that the normalized macroscopic strength in the nine granular assemblies exhibits a highly linear relationship with the normalized mesoscopic strength, which can be quantified through the following model

(9) $${\hat{\sigma }}_{d}=14.74{\hat{f}}_{\mathrm{mes}}-1.5$$

**Figure 14:**
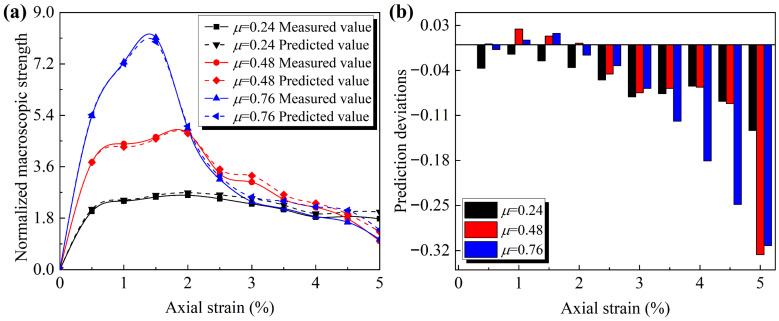
Predictive performance of normalized mesoscopic strength on normalized macroscopic strength throughout the loading phase: (**a**) predicted and measured ${\hat{\sigma }}_{d}$ with increasing axial strain; (**b**) trend of prediction errors with increasing axial strain.

The R-squared of Equation (9) is 0.97, indicating a strong linear correlation between the mesoscopic and macroscopic strength and that this relationship applies to particles with different morphologies. Figure 14 further demonstrates the applicability of this mapping relationship throughout an entire loading process (illustrated using three granular assemblies: *Ag* = 0.06, *μ* = 0.24; *Ag* = 0.06, *μ* = 0.48; *Ag* = 0.06, *μ* = 0.76).

It can be seen from Figure 14a that before reaching the peak deviatoric stress, Equation (9) demonstrates good predictive performance for the normalized macroscopic strength of all three granular assemblies. However, the prediction accuracy decreases during the residual stage. Additionally, it is observed that the prediction accuracy in the residual stage generally decreases with an increasing friction coefficient, as shown in Figure 14b. This dispersion in the residual stage is hypothesized to be related to the localized heterogeneity of the force transmission networks caused by the increase in porosity. Nevertheless, the overall trend of the predicted and measured macroscopic strength during the residual stage remains consistent.

Both Figure 13 and Figure 14 suggest that Equation (9) is an effective macro–mesoscopic strength correlation model. Compared with fabric-based approaches [54,55], this model establishes a clearer physical mapping between mesoscopic strength parameters and macroscopic strength. As a result, it provides a more transparent framework for interpreting the influencing mechanism of particle morphology and confining stress on the macroscopic strength of granular assemblies. In this model, the mesoscopic strength is determined by two mesoscopic properties: the mean inter-particle contact force and the mean coordination number. Both mesoscopic properties are governed by particle morphology. This model could explain the variations in macroscopic strength across granular assemblies with different morphologies. Using the three granular assemblies with a friction coefficient of 0.48 as a case study, the normalized mean inter-particle contact force increases significantly with increasing angularity (Figure 11), while the mean coordination number decreases sharply (Figure 12). These opposing trends in the two mesoscopic properties balance each other, resulting in only a small change in mesoscopic strength for the three particle assemblies. Consequently, this leads to minor differences in peak macroscopic strength (Figure 8).

The reasons for the variations in the mean inter-particle contact force and the mean coordination number in the above example are hypothesized as follows: increased angularity enhances the interlocking capacity of individual particles, leading to an increase in the normalized mean inter-particle contact force. Simultaneously, greater angularity increases void spaces among particles, resulting in a reduction in the mean coordination number. It should be noted that this conclusion is derived under the gradation conditions set in Figure 3. Considering the fine particle filling effect in a wide gradation, which can mitigate the reduction in coordination number caused by increased angularity, it is speculated that the strength of granular assemblies with high angularity may improve significantly under more optimal gradation conditions. However, due to the computational speed limitations of the DEM method, it is currently challenging to account for the influence of fine particle content while incorporating irregular particle morphologies.

#### 3.3.2. Mesoscale Influence Mechanisms of Confining Pressure

**Figure 15:**
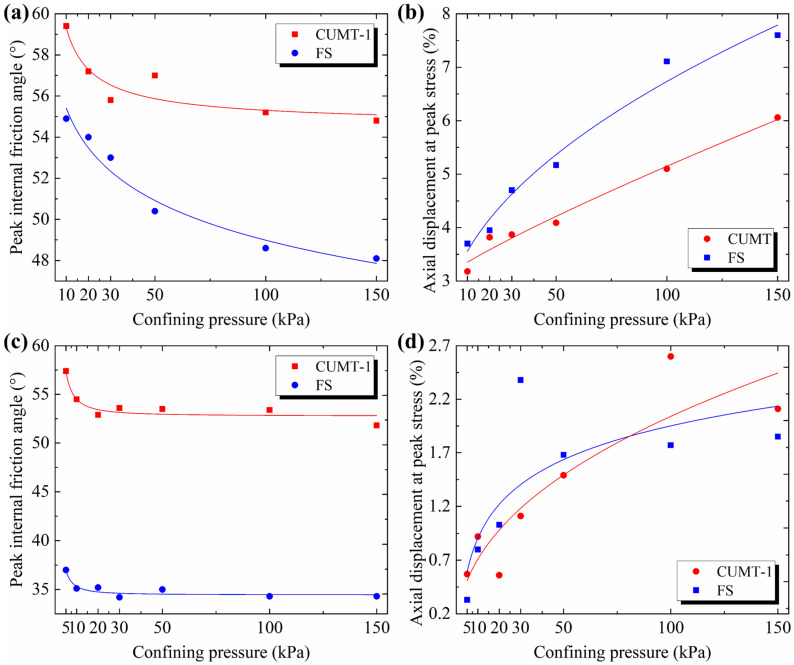
Influence of confining pressure on peak friction angle and axial strain at peak stress: (**a**) *φ*_peak_ vs. *σ*_3_ in triaxial tests; (**b**) the *ε*_1_ at peak stress vs. *σ*_3_ in triaxial tests; (**c**) *φ*_peak_ vs. *σ*_3_ in biaxial tests; (**d**) the *ε*_1_ at peak stress vs. *σ*_3_ in biaxial tests.

The main findings of this study regarding confining pressure are as follows: the peak friction angle of granular materials increases as confining pressure decreases, and the peak stress occurs earlier with decreasing confining pressure. These observed trends apply to both CUMT-1 lunar regolith simulant and Fujian standard sand, as quantified in Figure 15. In both triaxial and biaxial tests, the peak friction angles of the two materials exhibit an accelerated increase as confining pressure decreases, particularly below 10 kPa, as shown in Figure 15a,c. Besides, as confining pressure decreases, the axial strain corresponding to the peak stress of both materials shows an accelerated reduction, with the most significant acceleration also occurring below 10 kPa, as shown in Figure 15b,d.

**Figure 16:**
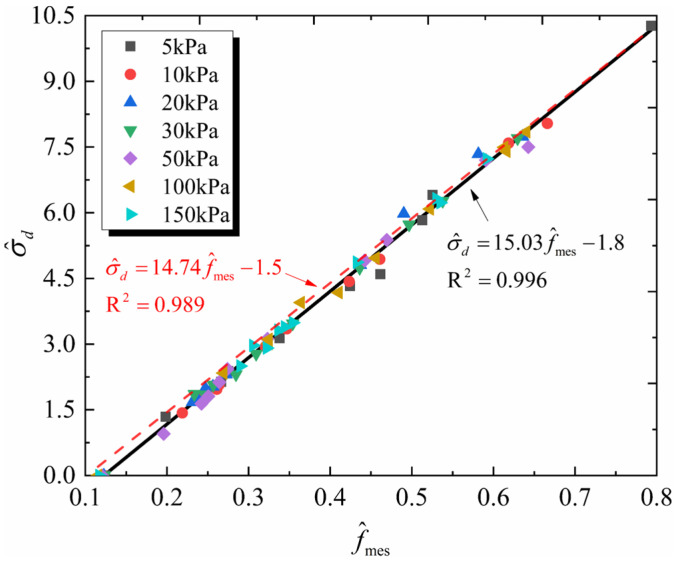
Distribution of normalized macroscopic and mesoscopic strengths under different confining pressures.

The macro–mesoscopic strength correlation model is used to reveal how confining pressure influences shear strength, with the CUMT-1 lunar regolith simulant as a case study. First, the model’s applicability under different confining pressures is verified, as shown in Figure 16.

The scatter points represent the distribution of normalized macroscopic and mesoscopic strengths of CUMT-1 lunar regolith simulant under various confining pressures. A total of 77 data points were collected, with 11 evenly selected from each confining pressure condition, covering the entire biaxial test process. The red dashed line shows the prediction effect of macroscopic strength under seven confining pressures by directly using Equation (9). It can be observed that the model effectively correlates the normalized macroscopic and mesoscopic strengths under different confining pressures, with a goodness of fit of 0.989. If the model is refitted using the data points under seven confining pressures, the model coefficients are slightly adjusted as follows

(10) $${\hat{\sigma }}_{d}=15.03{\hat{f}}_{\mathrm{mes}}-1.8$$

**Figure 17:**
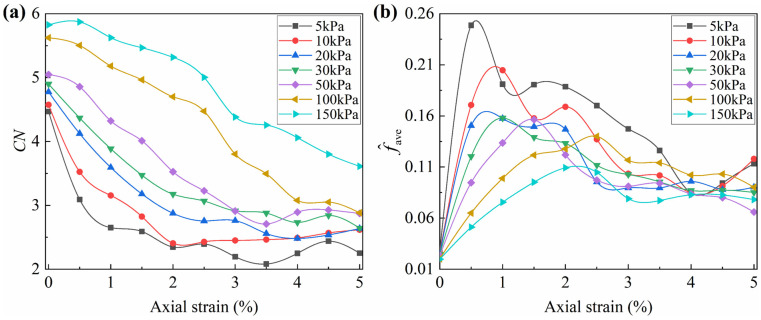
(**a**) Evolution trend of *CN* with axial strain; (**b**) evolution trend of ${\hat{f}}_{\mathrm{ave}}$ with axial strain.

The goodness of fit for Equation (10) improves to 0.996, suggesting that the proposed macro–mesoscopic strength correlation model is applicable under varying confining pressure conditions. Building on this, the variations in the mean coordination number and the normalized mean interparticle contact force are further analyzed to elucidate the mesoscopic influence mechanisms of confining pressure on shear strength. Figure 17 shows the variations in these two mesoscopic parameters with confining pressure.

Figure 17a shows that the mean coordination number decreases with decreasing confining pressure, which leads to a decrease in the normalized macroscopic strength. Meanwhile, the mean normalized interparticle contact force increases with increasing confining pressure, as illustrated in Figure 17b, which enhances the normalized macroscopic strength. Since Figure 15c shows that the peak friction angle increases with decreasing confining pressure, it can be inferred that the improvement in the normalized macroscopic strength is primarily driven by the increase in normalized interparticle contact force.

The reasons for the decrease in the mean coordination number and the increase in normalized mean interparticle contact force with decreasing confining pressure are further explored. Assuming that the confining pressure approaches zero, the sample will be in a state close to uniaxial compression. When the top and bottom walls compress the granular assembly, an instantaneous resistance stress is generated to act back on the walls, mainly due to the inertia of individual particles and the interlocking and frictional structures among particles. In this extreme case, the slight reaction force exerted on the top and bottom walls is the deviatoric stress. Since the confining pressure approaches zero, according to Equation (8), the normalized macroscopic strength tends toward infinity. Furthermore, since the confining pressure is close to zero, most particles not part of the main force chain will rapidly move toward the sides, leading to a sharp reduction in the mean coordination number. At the same time, particles within the main force chain gain slight interparticle contact forces as they resist the wall compression. According to Equation (7), the normalized mean interparticle contact force tends toward infinity.

In the above extreme case, the macroscopic deviatoric stress is generated by the destruction of the initial granular assembly structure, with confining pressure providing negligible lateral support. As a result, the peak strength will appear instantaneously and disappear instantaneously, and particles will rapidly move to the sides, leading to a noticeable volumetric expansion at the macroscopic level.

According to the above reasoning, the smaller the confining pressure, the closer the macro–mesoscopic mechanical behavior should approach the above extreme case. All the observed macro–mesoscopic trends in the biaxial tests strongly support the reasoning, as detailed below.

**Figure 18:**
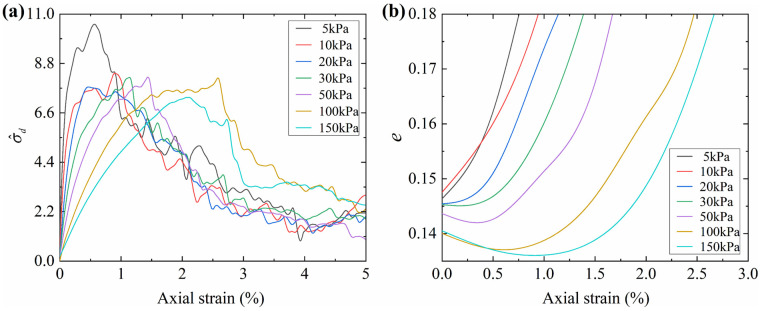
(**a**) Evolution trend of ${\hat{\sigma }}_{d}$ with axial strain; (**b**) evolution trend of *e* with axial strain.

(1) The mean coordination number and normalized mean interparticle contact force exhibit decreasing and increasing trends with decreasing confining pressure, respectively, as shown in Figure 17. Meanwhile, the normalized macroscopic strength continuously increases, as shown in Figure 15c.

(2) Both the normalized macroscopic peak strength and normalized peak interparticle contact force appear earlier with decreasing confining pressure, as shown in Figure 17b and Figure 18a. Meanwhile, the duration of their peak states decreases steadily.

(3) The phenomenon of increased volume expansion with decreasing confining pressure is observed from the evolution of the void ratio, as shown in Figure 18b. The void ratio at any loading stage was calculated using the following formula:

(11) $$e=\frac{h_{i}w_{i}}{h_{0}w_{0}}(1+e_{0})-1$$

where e_0_ represents the initial void ratio of the numerical sample, obtained through image binarization; h_0_ and w_0_ denote the initial height and width of the sample, respectively; h_i_ and w_i_ represent the height and width of the sample at any loading stage.

## 4. Conclusions and Prospects

The study combines physical experiments and discrete element analysis to investigate the macroscopic effects and mesoscopic mechanisms of irregular particle morphology and low geostatic stress on the shear strength of lunar regolith. The main conclusions are as follows.

1. A novel macro–mesoscopic strength correlation model has been proposed. It utilizes two mesoscopic indicators, normalized mean interparticle contact force and mean coordination number, to map the normalized macroscopic strength, demonstrating a goodness of fit better than 0.97 under various particle morphologies and confining pressure conditions.
2. The increase in both friction coefficient and angularity leads to a rise in interparticle contact force, thereby enhancing the peak friction angle, with the friction coefficient playing a dominant role. The increase in angularity simultaneously leads to a decrease in coordination number, which limits the improvement in peak shear strength, especially under poor particle gradations. In contrast, the particle morphology has less influence on the residual friction angle.
3. Decreased confining pressure reduces the coordination number while increasing the normalized interparticle contact force. The increased normalized interparticle contact force dominates the rise in the normalized mesoscopic strength, thereby causing the peak friction angle to increase. These trends apply to dry granular materials, regardless of particle morphology.

From an engineering perspective, the results of this study indicate that the low confining stress and high surface roughness of lunar surface regolith contribute to a higher internal friction angle. This may explain the high drilling resistance observed in lunar missions, while still providing favorable conditions for rover mobility. With increasing depth, the combined effects of rising confining pressure and reduced particle morphological complexity tend to decrease the internal friction angle, indicating a potential reduction in shear strength. This suggests that depth-dependent strength variation should be carefully considered in the design of foundations and subsurface structures. However, the increase in relative density with depth, as observed in Apollo missions, may counteract this trend. Future studies should therefore consider the coupled effects of these factors to improve the prediction of shear strength variation in subsurface lunar regolith.

Despite the insights obtained in this study, several limitations should be acknowledged. First, the experiments were conducted using lunar regolith simulants rather than real lunar soil, which may not fully capture the complex physical and mechanical properties of in situ lunar regolith. Second, although the tests were conducted under controlled confining pressures, potential membrane effects under low confining stress may influence the accuracy of the measured responses. Third, the DEM simulations involve certain simplifications in particle morphology and contact models, which may influence the accuracy of the mesoscopic responses. Future studies should consider incorporating more realistic particle representations and testing conditions to further improve the applicability of the findings.

## Figures and Tables

**Table 1:** Morphology quantification results of CUMT-1 lunar regolith simulant and Fujian standard sand under different particle sizes.

Particle Size (m)	CUMT-1 Lunar Regolith Simulant			Fujian Standard Sand		
	Aspect Ratio	Angularity	Texture	Aspect Ratio	Angularity	Texture
>2	0.726	0.0502	0.0195	0.743	0.0218	0.0083
1~2	0.697	0.0515	0.0184	0.741	0.0168	0.0072
0.5~1	0.728	0.0616	0.0190	0.822	0.0282	0.0081
0.25~0.5	0.739	0.0783	0.0187	0.753	0.0324	0.0086
0.1~0.25	0.701	0.0634	0.0181	0.762	0.0337	0.0098
0.05~0.1	0.712	0.0517	0.0179	0.687	0.0498	0.0103
Mean value	0.717	0.0600	0.0186	0.751	0.0305	0.0087

**Table 2:** Input parameters used in the DEM biaxial compression tests.

Parameter	Value	Parameter	Value
Damping ratio, *ζ*	0.7	Particle density, *ρ* (kg/cm^3^)	3.44 × 10^7^
Initial porosity, *n*	0.06	Particle-particle normal stiffness, *k_n_* (N/m)	7.5 × 10^7^
Particle friction coefficient, *μ_p_*	0.24–0.76	Particle-particle shear stiffness, *k_s_* (N/m)	5.0 × 10^7^
Wall frictional coefficient, *μ_w_*	0	Particle-wall normal stiffness, *k_n_* (N/m)	1.5 × 10^10^
Particle diameter, *d* (mm)	0.2–1.5	Particle-wall shear stiffness, *k_s_* (N/m)	1.0 × 10^10^
Specimen size (mm)	Φ 7.82 × 16 mm	Axial strain, *ε* (%/min)	0.975

**Table 3:** Strength parameters of CUMT-1 lunar regolith simulant and Fujian standard sand in triaxial tests.

Soil Type	*σ*_3_ (kPa)	*φ*_peak_ (°)	*φ*_residual_ (°)	*θ* (°)
CUMT-1	10	59.4	52.5	60.0
	20	57.2	49.3	54.7
	30	55.8	46.6	49.8
	50	57.0	45	47.8
	100	55.2	44.1	42.1
	150	54.8	41.2	33.7
FS	10	54.9	51.8	50.4
	20	54.0	48.0	44.0
	30	53.0	46.8	42.3
	50	50.4	44.2	37.2
	100	48.6	44.4	34.9
	150	48.1	44.3	31.3

**Table 4:** Strength parameters of CUMT-1 lunar regolith simulant and Fujian standard sand in biaxial tests.

Soil Type	*Ar*	*Ag*	*Te* (*μ*)	*σ*_3_ (kPa)	*φ*_peak_ (°)	*φ*_residual_ (°)
Numerical particles	0.5–0.91	0	0.24	50	35.9	24.7
		0	0.48	50	43.4	22.5
		0	0.76	50	50.1	26.4
		0.03	0.48	50	45.6	29.0
		0.03	0.76	50	52.6	27.3
		0.06	0.24	50	35.2	29.3
		0.06	0.48	50	44.9	29.1
CUMT-1	0.5–0.91	0.06	0.76	5	57.4	30.0
				10	54.5	28.8
				20	52.9	27.8
				30	53.6	31.0
				50	53.5	27.7
				100	53.4	38.4
				150	51.8	37.7
FS	0.5–0.91	0.03	0.24	5	37.0	30.0
				10	35.1	29.9
				20	35.2	28.2
				30	34.2	28.4
				50	35.0	28.4
				100	34.3	28.4
				150	34.3	28.1

## Data Availability

The original contributions presented in this study are included in the article. Further inquiries can be directed to the corresponding author.
